# Circular RNA expression profile and potential function of hsa_circRNA_101238 in human thoracic aortic dissection

**DOI:** 10.18632/oncotarget.18998

**Published:** 2017-07-05

**Authors:** Meisheng Zou, Chixiong Huang, Xinzhong Li, Xiang He, Yanmei Chen, Wangjun Liao, Yulin Liao, Jie Sun, Ze Liu, Lintao Zhong, Jianping Bin

**Affiliations:** ^1^ Department of Cardiology, State Key Laboratory of Organ Failure Research, Nanfang Hospital, Southern Medical University, Guangzhou, China; ^2^ Wards of Cadres, Guangzhou General Hospital of Guangzhou Military Region, Guangzhou, China; ^3^ Department of Oncology, Nanfang Hospital, Southern Medical University, Guangzhou, China; ^4^ Department of Cardiology, Zhongshan Hospital, Sun Yat-Sen University, Zhongshan, China

**Keywords:** thoracic aortic dissection, circular RNA, microarray, biomathematics, Pathology Section

## Abstract

**Objective:**

To assess the circular RNAs (circRNAs) expression profile and explore the potential functions in human thoracic aortic dissection (TAD).

**Methods:**

The differentially expressed circRNAs profiles of the aortic segments between human type A TAD patients (*n*=3) and age-matched normal donors (NA; *n*=3) were analyzed using the Arraystar human circRNAs microarray. Quantitative real-time PCR was used to validate the expression pattern of circRNAs, parental genes, and hsa-miR-320a; Western blotting confirmed MMP9 expression with additional samples. Bioinformatic tools including network analysis, Gene ontology, and KEGG pathway analysis were utilized.

**Results:**

Among 8,173 detected circRNA genes, 156 upregulated and 106 downregulated significantly in human TAD as compared to NA (P£0.05). Quantitative real-time PCR showed an elevated expression of the upregulated hsa_circRNA_101238, hsa_circRNA_104634, hsa_circRNA_002271, hsa_circRNA_102771, hsa_circRNA_104349, COL1A1, and COL6A3 and reduced of the downregulated hsa_circRNA_102683, hsa_circRNA_005525, hsa_circRNA_103458, and FLNA. Gene ontology analysis revealed that the parental genes favored several pathological processes, such as negative regulation of cell proliferation and extracellular matrix organization. The circRNA-miRNA co-expression network predicted that 33 circRNAs might interact with at least one target miRNAs altered in TAD. KEGG pathway analysis revealed that 28 altered miRNAs were enriched on focal adhesion and vascular smooth muscle contraction. The hsa_circRNA_101238-miRNA-mRNA network indicated the highest degree of hsa-miR-320a. Quantitative real-time PCR and Western blot manifested the low expression of hsa-miR-320a and high of MMP9 in human TAD tissues, respectively.

**Conclusions:**

This study revealed hundreds of differentially expressed circular RNAs in human TAD, suggesting that hsa_circRNA_101238 might inhibit the expression of hsa-miR-320a and increase that of MMP9 in TAD.

## INTRODUCTION

Thoracic aortic dissection (TAD), a life-threatening vascular disease, is characterized by the separation of aortic wall layers. The key pathological feature of TAD is the disruption of the aortic extracellular matrix (ECM) and depletion of aortic smooth muscle cells (SMCs) [1]. Despite advances in open or endovascular surgical techniques and medical therapy over the years, the overall morbidity and mortality of TAD remains high [2]. Owing to such challenging clinical dilemma, the molecular mechanisms responsible for initiating the dissection are not yet elucidated.

Hitherto, studies are primarily focused on mutations in several categories of TAD-associated protein-coding genes such as ECM genes (*FBN1*, *LOX*, *COL3A1*) [3-5] and SMCs genes (*ACTA2*, *MYH11*, *FLNA*) [6-8], which partially elucidated the general pathological processes of TAD. Recently, non-coding RNAs (ncRNAs) have been reported to play critical roles in aortic neurysmal disease[9]. Therefore, excluding the protein functions, efforts should be emphasized on understanding the non-protein functions in the progression of TAD and investigating the functions of ncRNAs that have received extensive attention due to their differential expression in TAD and normal aorta tissue [10, 11]. MicroRNAs (miRNAs), the most studied ncRNAs, have been shown to contribute towards the altered ECM and SMCs in an aortic aneurysm and dissection [9, 12].

Compared with miRNAs, Circular RNAs (circRNAs) are another new type of ncRNAs that are formed covalently closed loop structures [13]and widely expressed in human cells[14]. Most circRNAs are competing endogenous RNAs(ceRNA) and show tissue and developmental stage-specific expression[15]. It was reported that circRNAs could regulate the expression of parental gene [16, 17] or function as miRNAs sponges [18, 19]. It was demonstrated that circRNAs are involved in the progression of several types of diseases, such as cancer [20] and Alzheimer’s disease [21]. Moreover, some evidence postulates that circRNAs may be involved in vascular function. For example, 4464 tissue-specific circRNAs were detected in the human normal aorta. [22] Also, a recent study showed that circRNAs were aberrantly expressed in hypoxia in human umbilical endothelial cells and exhibited a physiological function *in vitro* [23]. In addition, circRNA cANRIL may be correlated with atherosclerotic vascular disease risk *in vitro* [24, 25]. Nonetheless, these circRNAs studies related to vascular function have been conducted on the cellular or normal blood vessel level. However, to the best of our knowledge, no study has been reported on circRNAs in human TAD tissue.

In the present study, we used circRNA microarray to acquire circRNA profiles in human TAD tissues as compared to normal tissues. Subsequently, we performed biomathematical analysis to explore the potential functions in human TAD. Thus, these data would lay a foundation for future investigations on the molecular functions of circRNAs in human TAD.

## RESULTS

### General profiles of circRNA microarray and validation of differentially expressed circRNAs using qRT-PCR

**Figure 1:**
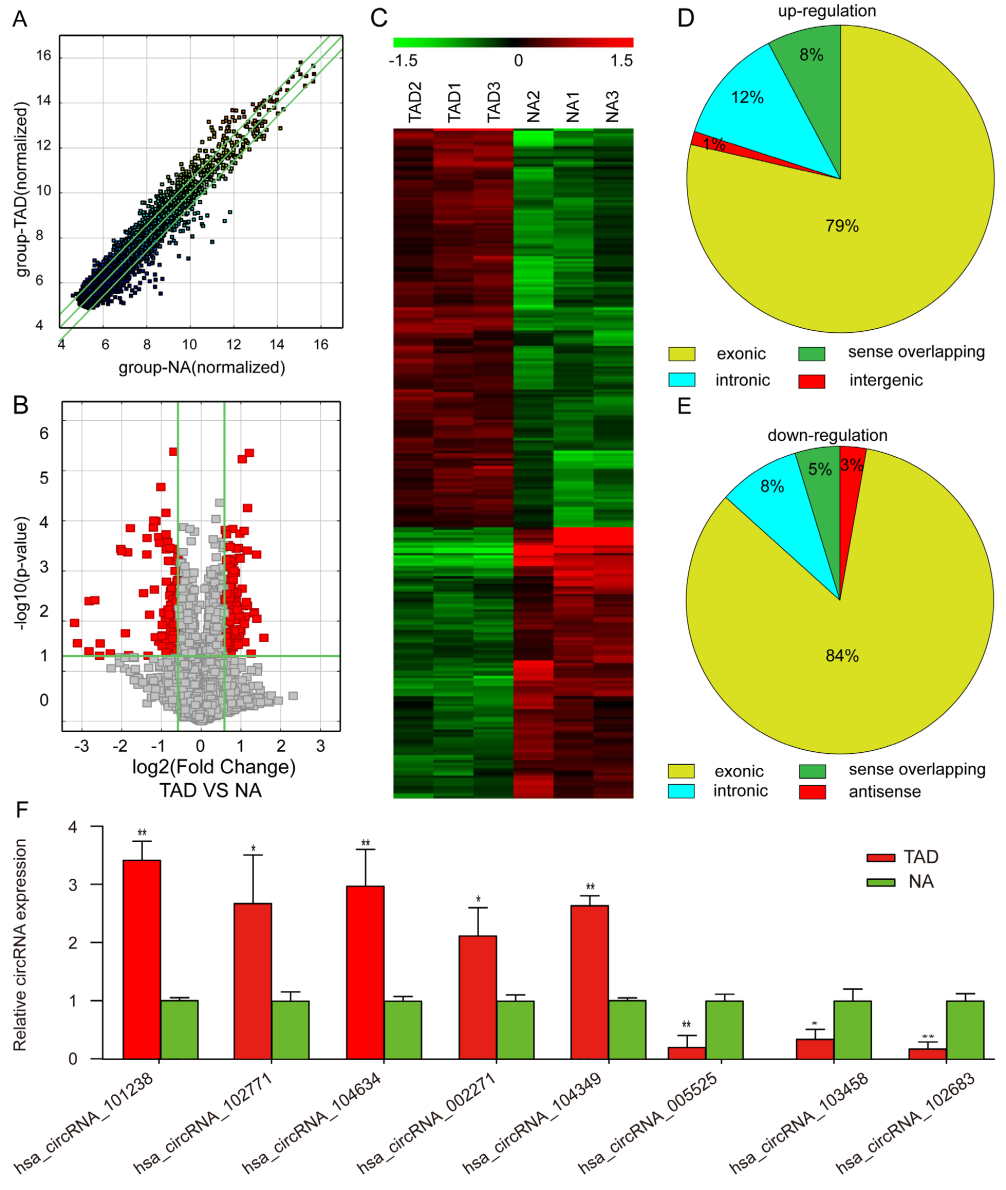
Differential expression of circRNAs in aortic tissues **A.** Scatter plots are used to evaluate the difference in the expression of circRNAs between experimental and control groups. The values plotted on X and Y axes are the averaged normalized signal values of each group (log_2_ scaled). The middle green line indicates no difference between the two groups. The flanking green lines represent 1.5-fold changes. The circRNAs above the top green line and below the bottom green line indicate >1.5-fold changes between the two groups. **B.** Volcano plots are used for visualizing differential expression between the two different conditions. The vertical lines correspond to 1.5-fold (log_2_ scaled) increase and decrease, respectively, and the horizontal line represents *P* = 0.05 (−log_10_ scaled). The red points in the plot represent the differentially expressed circRNAs with statistical significance. **C.** The hierarchical clustering of partial differentially expressed circRNAs. ‘Red’ indicates high relative expression, and ‘green’ indicates low relative expression. **D.** Upregulated circRNAs. **E.** Downregulated circRNAs. **F.** Validation of the differential expression of eight circRNAs. The data in the figures represents the average ± SD. ^*^ *P* < 0.05, and **P < 0.01 by Student’s t-test.

A total of 8,173 circRNAs were detected by Arraystar human circRNA Microarray (Supplemental Table S5). The differentially expressed circRNAs were displayed through fold-change filtering (Figure 1A). The volcano plot filter identified the differentially altered circRNAs with statistical significance between human TAD and NA samples (Figure 1B). Hierarchical clustering revealed the circRNA expression in human TAD and NA samples (Figure 1C). As a result, 262 circRNAs were dysregulated in patients with TAD as compared to the NA tissue (fold-change≥1.5, P£0.05), among which 156 circRNAs were upregulated while 106 were downregulated (Supplemental Table S6). Thus, we summarized the classification of dysregulated circRNAs as described below. Among the upregulated circRNAs, there were 2 intergenic, 12 sense overlapping, 19 intronic, and 123 exonic(Figure 1D). Among the downregulated circRNAs, 3 were antisense, 5 sense overlapping, 9 intronic, and 89 exonic (Figure 1E). Moreover, to validate the circRNA microarray profiling expression results, qRT-PCR revealed five upregulated circRNAs (hsa_circRNA_101238, hsa_circRNA_104634, hsa_circRNA_002271, hsa_circRNA_102771, and hsa_circRNA_104349) and three downregulated circRNAs (hsa_circRNA_102683, hsa_circRNA_005525, and hsa_circRNA_103458) as shown in Figure 1F. The data indicated that qRT-PCR results were in agreement with the microarray analysis regarding the expression levels of the eight circRNAs.

### GO analysis and validation of the dysregulated circRNAs

**Figure 2:**
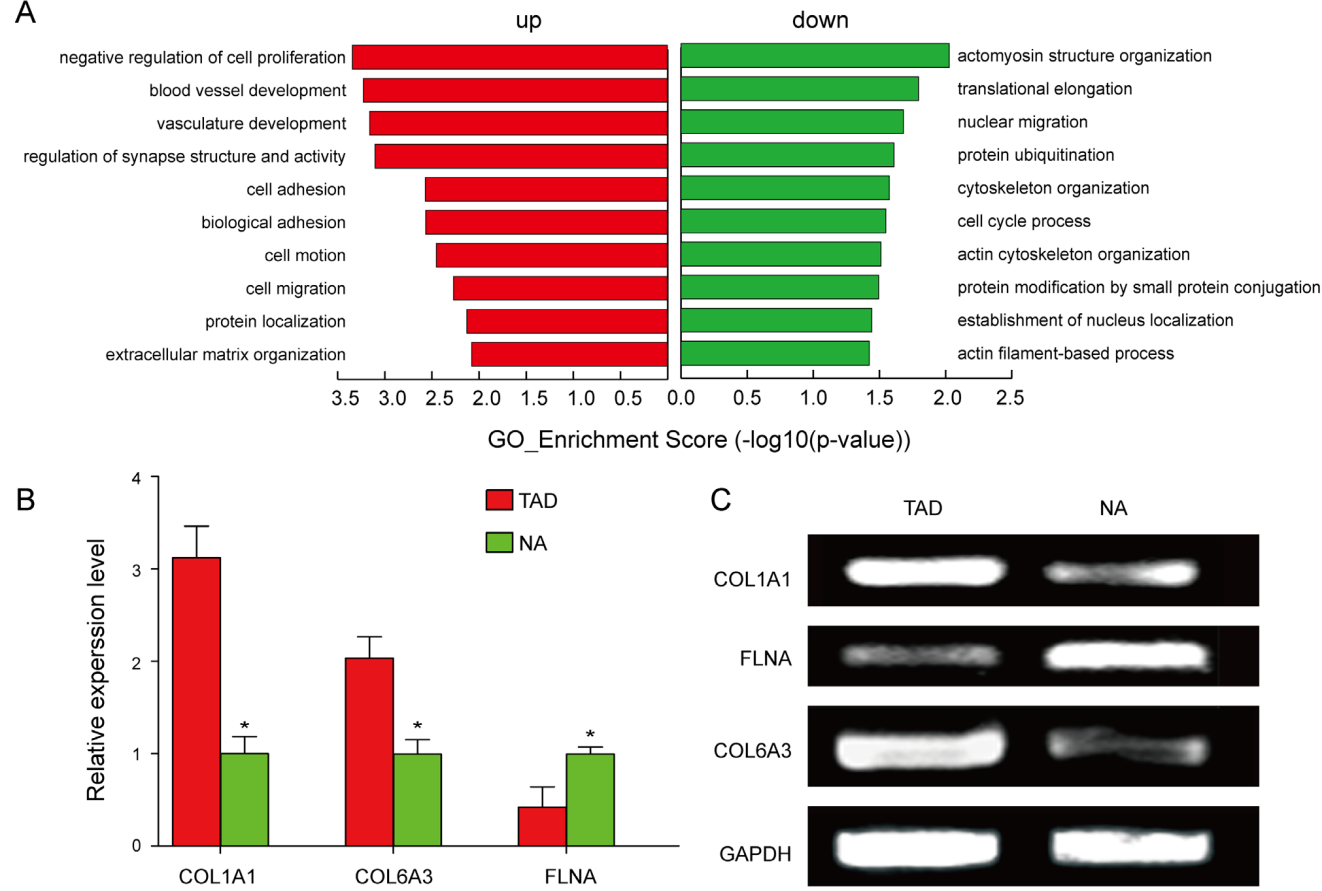
GO analysis and validation of the dysregulated circRNAs gene symbols **A.** GO annotation of the linear counterparts of upregulated and downregulated circRNAs with top 10 enrichment score encompassing the domains of physiological processes. Both up and down regulated circRNAs are significantly altered with ≥1.5 fold-change and P£0.05. **B.**, **C.** Validation of the upregulated parental genes, *COL1A1* and *COL6A3*, and the down-regulated parental gene, *FLNA*.

In order to explore whether circRNAs regulate the parental gene transcription, GO analysis of the genes producing differently expressed circRNAs was performed. Compared to the NA tissues, the data revealed that the gene expression profile of linear counterparts of differentially over-expressed circRNAs in TAD group favored regative regulation of cell proliferation and ECM organization (Figure 2A). On the other hand, the GO enrichment analysis on the downregulated transcripts of TAD showed that closely related GO terms were actomyosin structure organization, actin cytoskeleton organization, and actin filament-based process. (Figure 2A). In addition, we also validated the upregulated parental genes, *COL1A1* and *COL6A3*, and the downregulated parental gene, *FLNA*, between TAD and NA tissues (Figure 2B, 2C).

### Construction of the circRNA-miRNA interaction network and functional annotations for target genes of altered miRNAs

**Figure 3:**
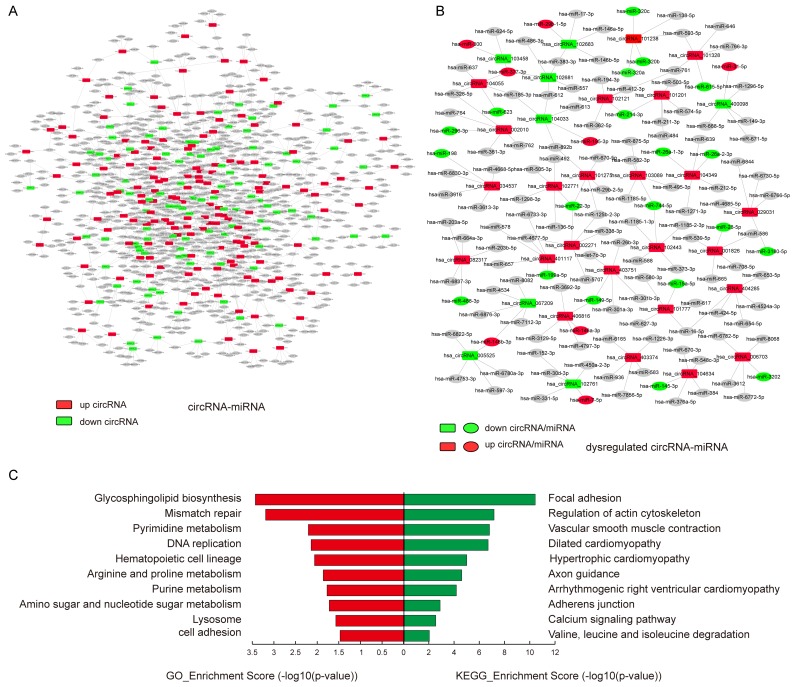
The circRNA-miRNA co-expression network and functional annotations for target genes of altered miRNAs **A.** The panorama network comprising 156 upregulated circRNAs (red rectangle nodes), 106 downregulated circRNAs (green rectangle nodes), and their top 5 target miRNAs. **B.** A magnified network consists of 25 upregulated circRNAs(red rectangle nodes) and their 24 target downregulated miRNAs (green oval nodes), eight downregulated circRNAs (green rectangle nodes) and their seven target upregulated miRNAs (red oval nodes). **C.** Functional annotations for the target genes of altered miRNAs.

To elucidate the function of circRNAs, the basic circRNA-miRNA connectivity was established by TargetScan coupled with miRanda. All the differentially expressed circRNAs were predicted according to the complementary miRNA matching sequence; the targeted miRNAs were ranked according to the mirSVR scores, thereby identifying the 5 highest ranking candidates (“Top 5”) for further analysis (Figure 3A). In the panorama network, the red rectangle nodes represent upregulated circRNAs and the blue rectangle nodes represent downregulated circRNAs. Furthermore, 25 upregulated and eight downregulated circRNAs (Table 1), which have at least one predicted target miRNA modified in TAD were selected for further study. A network of circRNA-miRNA interaction between the 33 circRNAs described above and their target miRNAs was constructed using Cytoscape (Figure 3B). In the magnified network, rectangle nodes represent circRNAs and oval nodes represent their target miRNAs. Red color and blue color represents up and down regulation respectively. The co-expression network reveals that most of the 33 circRNAs predicted one or two TAD-associated miRNAs. Only hsa_circRNA_101238 established interactions with the three altered miRNAs (hsa-miR-320a, hsa-miR-320b, and hsa-miR-320c). Then, we annotated target genes of the 28 altered miRNAs, which showed a strong correlation with focal adhesion, regulation of actin cytoskeleton, and vascular smooth muscle contraction (Figure 3C).

**Table 1:** circRNAs that have at least one predicted target miRNA modified in TAD

circRNA	P-value	FC	Regulation	MRE1	MRE2	MRE3	MRE4	MRE5
hsa_circRNA_082317	4.414E-06	2.316	up	**hsa-miR-488-3p**	hsa-miR-203a-5p	hsa-miR-664a-3p	hsa-miR-203b-5p	hsa-miR-6837-3p
hsa_circRNA_401117	0.002	1.866	up	hsa-miR-4677-5p	hsa-miR-6733-3p	**hsa-miR-199a-5p**	hsa-miR-578	hsa-miR-657
hsa_circRNA_101238	0.005	1.841	up	**hsa-miR-320b**	**hsa-miR-320a**	hsa-miR-138-5p	hsa-miR-593-5p	**hsa-miR-320c**
hsa_circRNA_104634	0.008	1.779	up	hsa-miR-384	hsa-miR-670-3p	hsa-miR-548c-3p	**hsa-miR-145-3p**	hsa-miR-376a-5p
hsa_circRNA_001826	0.001	1.772	up	hsa-miR-708-5p	**hsa-miR-28-5p**	hsa-miR-586	hsa-miR-539-5p	hsa-miR-665
hsa_circRNA_002271	0.001	1.713	up	hsa-miR-4677-5p	**hsa-miR-22-3p**	hsa-miR-338-3p	hsa-miR-125b-2-3p	hsa-miR-5707
hsa_circRNA_102771	0.001	1.712	up	hsa-miR-492	hsa-miR-1298-3p	**hsa-miR-22-3p**	hsa-miR-505-3p	hsa-miR-136-5p
hsa_circRNA_101201	0.023	1.709	up	hsa-miR-503-5p	hsa-miR-574-5p	hsa-miR-484	hsa-miR-211-3p	**hsa-miR-214-3p**
hsa_circRNA_104349	0.004	1.697	up	hsa-miR-212-5p	**hsa-miR-26a-1-3p**	**hsa-miR-26a-2-3p**	hsa-miR-639	hsa-miR-1271-3p
hsa_circRNA_101328	0.016	1.694	up	**hsa-miR-615-5p**	hsa-miR-761	hsa-miR-593-5p	hsa-miR-766-3p	hsa-miR-646
hsa_circRNA_006703	0.046	1.686	up	**hsa-miR-3202**	hsa-miR-6782-5p	hsa-miR-8068	hsa-miR-3612	hsa-miR-6772-5p
hsa_circRNA_029031	0.004	1.682	up	hsa-miR-6766-5p	hsa-miR-6844	hsa-miR-6730-5p	hsa-miR-4685-5p	**hsa-miR-3180-5p**
hsa_circRNA_103089	0.001	1.656	up	hsa-miR-582-3p	hsa-miR-1185-5p	hsa-miR-875-5p	**hsa-miR-744-5p**	hsa-miR-495-3p
hsa_circRNA_102121	0.001	1.639	up	hsa-miR-412-3p	**hsa-miR-214-3p**	hsa-miR-194-3p	hsa-miR-761	hsa-miR-362-5p
hsa_circRNA_403751	0.009	1.631	up	hsa-let-7c-3p	**hsa-miR-149-5p**	hsa-miR-26b-3p	hsa-miR-301b-3p	hsa-miR-301a-3p
hsa_circRNA_101565	0.004	1.616	up	hsa-miR-617	hsa-miR-627-3p	hsa-miR-16-5p	hsa-miR-580-3p	**hsa-miR-15a-5p**
hsa_circRNA_104055	0.004	1.579	up	hsa-miR-637	hsa-miR-328-5p	**hsa-miR-623**	hsa-miR-377-3p	hsa-miR-185-3p
hsa_circRNA_404285	0.026	1.577	up	hsa-miR-16-5p	hsa-miR-4524a-3p	**hsa-miR-424-5p**	hsa-miR-653-5p	hsa-miR-654-5p
hsa_circRNA_403374	0.003	1.555	up	**hsa-miR-936**	hsa-miR-6165	hsa-miR-1226-3p	hsa-miR-583	hsa-miR-7856-5p
hsa_circRNA_406816	0.017	1.551	up	hsa-miR-3692-3p	hsa-miR-4797-3p	**hsa-miR-149-5p**	hsa-miR-146a-3p	hsa-miR-3129-5p
hsa_circRNA_102443	0.014	1.548	up	hsa-miR-588	**hsa-miR-744-5p**	hsa-miR-373-3p	hsa-miR-1185-2-3p	hsa-miR-1185-1-3p
hsa_circRNA_034537	0.016	1.548	up	hsa-miR-3613-3p	**hsa-miR-198**	hsa-miR-4668-5p	hsa-miR-6830-3p	hsa-miR-3916
hsa_circRNA_002010	0.007	1.547	up	hsa-miR-328-5p	**hsa-miR-296-3p**	hsa-miR-764	hsa-miR-762	hsa-miR-361-3p
hsa_circRNA_101777	0.020	1.541	up	**hsa-miR-214-3p**	**hsa-miR-744-5p**	hsa-miR-25-5p	hsa-miR-205-5p	hsa-miR-450b-3p
hsa_circRNA_101275	0.020	1.525	up	hsa-miR-892b	hsa-miR-29b-2-5p	**hsa-miR-22-3p**	hsa-miR-670-5p	hsa-miR-125b-2-3p
hsa_circRNA_102683	0.007	2.459	down	hsa-miR-146b-5p	hsa-miR-146a-5p	hsa-miR-383-3p	hsa-miR-17-3p	**hsa-miR-29b-1-5p**
hsa_circRNA_005525	0.001	2.071	down	hsa-miR-6780a-3p	hsa-miR-4753-3p	hsa-miR-6822-5p	hsa-miR-597-3p	**hsa-miR-146b-3p**
hsa_circRNA_102681	0.019	1.978	down	hsa-miR-383-3p	hsa-miR-486-3p	hsa-miR-557	hsa-miR-612	**hsa-miR-377-3p**
hsa_circRNA_103458	0.011	1.972	down	hsa-miR-624-5p	**hsa-miR-300**	**hsa-miR-377-3p**		
hsa_circRNA_400098	4.165E-06	1.609	down	hsa-miR-671-5p	**hsa-miR-31-5p**	hsa-miR-1296-5p	hsa-miR-668-5p	hsa-miR-149-3p
hsa_circRNA_102761	0.003	1.576	down	**hsa-miR-7-5p**	hsa-miR-152-3p	hsa-miR-450a-2-3p	hsa-miR-30d-3p	hsa-miR-331-5p
hsa_circRNA_067209	0.013	1.573	down	hsa-miR-8082	**hsa-miR-146b-3p**	hsa-miR-6876-3p	hsa-miR-4534	hsa-miR-7112-3p
hsa_circRNA_104033	0.001	1.511	down	hsa-miR-612	hsa-miR-892b	hsa-miR-185-3p	**hsa-miR-195-3p**	hsa-miR-613

MRE: miRNA response element.

### Prediction of hsa_circRNA_101238-targeted miRNA-mRNA network and validation of the expression of hsa-miR-320a and MMP9

**Figure 4:**
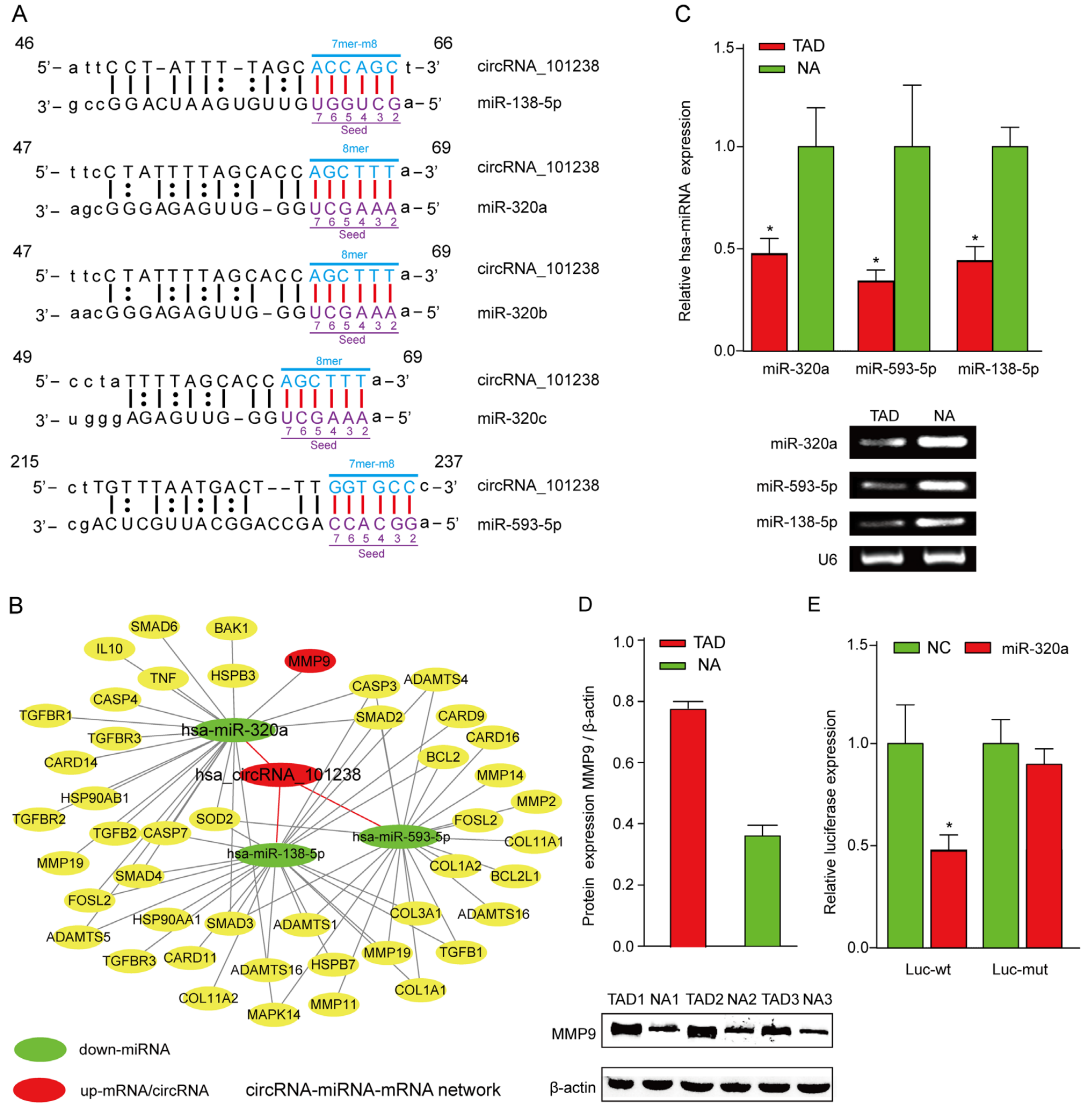
The biomathematically predicted hsa_circRNA_101238-targeted miRNA-mRNA network based on sequence-pairing prediction and validation for the expression of hsa-miR-320a and MMP9 **A.**hsa_circRNA_101238 and its top 5 predicted miRNA targets. **B.** 5 miRNAs and 46 genes were targeted in the present hsa_circRNA_101238-miRNA-mRNA network. In this figure, hsa-miR-320a exhibited the highest degree, followed by hsa-miR-138-5p, hsa-miR-593-5p, hsa-miR-320b, and hsa-miR-320c. **C.** qRT-PCR analysis shows the expression of hsa-miR-320a, hsa-miR-138-5p and hsa-miR-593-5p in the TAD tissues as compared to the normal controls. *P<0.05, *n* = 3. **D.** Western blot analysis shows the level of MMP9 protein in the TAD tissues as compared to NA. β-actin is an internal control. **P < 0.01, *n* = 3. **E.** Luciferase reporter analysis of either wild-type or mutant hsa-miR-320a activity. hsa_circRNA_101238 was co-transfected with the wild-type or mutant vector. The presented values are the mean ± SEM of 3 different preparations *P < 0.05.

In order to explore the potent competing endogenous RNA (ceRNA) molecules of circRNAs, the circRNA- miRNA-mRNA network was predicted. According to the initial microarray data analysis, the “Top 5” predicted miRNA targets of hsa_circRNA_101238 were hsa-miR-320b, hsa-miR-320a, hsa-miR-138-5p, hsa-miR-593-5p, and hsa-miR-320c (Figure 4A) out of which, three were downregulated in the TAD aortic specimen [4]. Therefore, hsa_circRNA_101238 was selected for further analysis. The genomic locus of hsa_circRNA_101238 is on chromosome 13, and the predicted sequence of the best linear transcript is NM_175854. Presuming that hsa_circRNA_101238 is an upstream molecular sponge for its target miRNAs, we predicted a hsa_circRNA_101238-miRNA-mRNA network using TargetScan and miRanda. Through target gene prediction, 46 genes (Supplementary Table S7) out of the 5 miRNAs mentioned above were collected. Each mRNA was experimentally validated by at least one of the following methods including qPCR, Western blot, and microarray. Then, the integrated hsa_circRNA_101238-miRNA-mRNA network was determined (Figure 4B) in parallel with the analysis of the effect of hsa_circRNA_101238 on MMP9 expression by functioning as a ceRNA. Furthermore, we examined the expression levels of hsa-miR-320a, hsa-miR-138-5p, hsa-miR-593-5p and MMP9 in the TAD tissues as compared to the NA. The low expression of hsa-miR-320a, hsa-miR-138-5p and hsa-miR-593-5p was validated in the TAD tissues using qRT-PCR (Figure 4C), whereas the high expression of MMP9 in the TAD tissues was detected by Western blotting (Figure 4D). The luciferase reporter was constructed to determine whether hsa_circRNA_101238 can directly target the hsa-miR-320a. The luciferase signal of hsa-miR-320a was suppressed by the wild-type hsa_circRNA_101238, whereas the luciferase signal of hsa-miR-320a was not affected by the mutant hsa_circRNA_101238 (Figure 4E).

## DISCUSSION

In the present study, we found that the differential expression of circRNAs between human TAD tissues and normal control tissues was universal. With GO analysis of their parental genes and construction of the circRNA-miRNA co-expression network, the dysregulated circRNAs could be involved in the pathological processes of TAD. Importantly, the upregulated hsa_circRNA_101238 might inhibit the expression of hsa-miR-320a by acting as an miRNA sponge, and in turn, increasing the expressions of several TAD-associated mRNAs, especially MMP9.

Increasing evidence indicates that circRNAs expression has spatio-temporal features. 1,903 circRNAs were identified in mouse tissues [18]. The expression abundance of circRNAs in 13 different human tissues were compared [26]. We identified 8173 circRNAs in human thoracic aortas as a circRNA-abundant tissue. Traditionally, circRNAs mainly arise from back-spliced exons [27, 28]. In accordance with previous researches, we found nearly 79% upregulated circRNAs belong to exonic and 84% downregulated circRNAs belong to exonic.

The dysregulated circRNAs may regulate the expression of parental genes and play a role in the pathological processes of diseases. With GO analysis, our findings demonstrated that some dysregulated circRNAs might participate in the pathological processes of TAD, such as negative regulation of cell proliferation, ECM organization, actomyosin structural organization, actin cytoskeleton organization, and actin filament-based process. For example, hsa_circRNA_051799, the most upregulated circRNA in the current study, was spliced by BAX, which is elevated in apoptotic SMC [29, 30], indicating that hsa_circRNA_051799 might negative regulation of the cell proliferation. In addition, the upregulated circRNAs, hsa_circRNA_081069, hsa_circRNA_102121, and hsa_circRNA_058819 were derived from COL1A2, COL1A1, and COL6A3, respectively. The expression of COL1 and COL6, which are the vital components of the ECM, is enhanced in TAD [31]. Therefore, hsa_circRNA_081069, hsa_circRNA_102121, and hsa_circRNA_058819 might promote the ECM organization. Moreover, the downregulated circRNAs, hsa_circRNA_405537, and hsa_circRNA_064175, were spliced from MYH10 and ARPC4. MYH10 and ARPC4, members of actomyosin and actin, were not explored extensively in TAD; however, MYH11 and ACTA2, similar to MYH10 and ARPC4, were demonstrated to activate the phenotype of SMCs [6, 7]. Thus, it was possible to alter the actomyosin structure organization and actin cytoskeleton organization in TAD. Also, the downregulated circRNAs, hsa_circRNA_105039, hsa_circRNA_105038, hsa_circRNA_092022, and hsa_circRNA_105040 were spliced from filamin A (FLNA). Reportedly, FLNA mutations are associated with TAD [8]. A comparative microarray study of gene expression profiles in TAD showed a decreased expression of FLNA [32]. FLNA is a well-known classic and crucial member in actin filament-based process. Thus, hsa_circRNA_105039, hsa_circRNA_105038, hsa_circRNA_092022, and hsa_circRNA_105040 might alter the actin filament-based process involved in TAD by regulating FLNA.

Intriguingly, the circRNA-miRNA co-expression network constructed in our study postulated that circRNAs might be essential for the development and progression of TAD as they harbor the miRNA binding sites. Our results also demonstrated that some upregulated circRNAs might inhibit the expression of several downregulated TAD-associated miRNAs. For instance, the upregulated hsa_circRNA_101238 interacted with hsa-miR-320a, hsa-miR-320b, hsa-miR-320c, which were reportedly downregulated in a miRNAs expression profiling analysis of TAD aortic specimen [4]. The upregulated hsa_circRNA_104634 and hsa_circRNA_104349 interacted with hsa-miR-145-3p and hsa-miR-26a-3p, respectively that have been demonstrated to increase SMC phenotype or apoptosis [23-24]. In addition, we found that some downregulated circRNAs might inhibit the expression of several upregulated TAD-associated miRNAs. For example, the downregulated hsa_circRNA_102683 and hsa_circRNA_104033 inhibited hsa-miR-29b-1-5p and hsa-miR-195-3p, respectively, which has been shown to target the aortic wall apoptosis and ECM degradation or stimulation of the collagen remodeling [25-26]. Furthermore, we used the KEGG pathway analysis to annotate the predicted genes of 28 altered miRNAs. According to our annotation, the altered miRNAs associated with focal adhesion, vascular smooth muscle contraction pathway, and regulation of actin cytoskeleton, and that are involved in TAD [9, 30] Hence, together with the interactions of TAD-associated miRNA, those circRNAs may be critical for the development and progression of TAD.

Additionally, the potential function of hsa_circRNA_101238 has been analyzed for the first time in human TAD. The circRNAs could function as miRNA sponges and might compete against other endogenous RNAs. ciRS-7 may act as an miR-7 sponge to inhibit miR-7 activity, resulting in an increased expression of miR-7 target genes [16]. Similarly, SRY was another miRNA sponge that harbored 16 binding sites for miR-138 [13]. Under the circRNA-miRNA-mRNA network, we inferred that the upregulated hsa_circRNA_101238 and the downregulated hsa-miR-320a interaction resulted in an elevated expression of several TAD-associated mRNAs such as MMP9. This speculation was partially supported by our PCR and Western blot evidence. We found that hsa_circRNA_101238 and MMP9 were highly expressed in human TAD tissues as compared to NA tissues, whereas hsa-miR-320a was lowly expressed. A previous study showed that the increased expression of MMP9 in human TAD cases was in agreement with our results [33]. In addition, MMP9 was directly declined by miR-320 via its 3′ UTR target sequences as assessed by luciferase reporter gene assays [34]. Also, the expression of miR-320a that targets MMP9 mRNA, was significantly decreased in B cells in patients with multiple sclerosis and may contribute towards an increased blood-brain barrier permeability and neurological disability [35]. Therefore, hsa_circRNA_101238 may initially inhibit the expression of hsa-miR-320a, subsequently increasing the expression of the target genes, and finally participating in the pathogenesis of TAD.

There are several limitations in this study. There are several limitations in this study. First, the data in this study is based on a small sample size. Future studies should endeavor to recruit larger number of TAD samples to confirm our results. Second, the sample collection time in our study was all at day. We will collect the sample at night and analysis how circadian rhythm affected the circRNA expression. Third, the study of circRNAs in TAD has just started, and the functional analysis is imperfect. Further functional studies should be done to improve this shortcoming in the future.

In conclusion, we found hundreds of differentially expressed circular RNAs in patients with TAD as compared to the NA tissue. Dysregulated circRNAs in human TAD tissue were associated with the pathological processes of disruption of the aortic ECM and depletion of aortic SMCs. Differentially expressed circular RNAs may play essentital roles in the development of TAD by regulating the expression of their parental genes or acting as miRNA sponges. Importantly, circRNA_101238-miR-320a-MMP9 axis may be involved in the pathogenesis of TAD.

## MATERIALS AND METHODS

### Patients and specimens

This study was conducted in accordance with the Declaration of Helsinki and was approved by the Medical Ethics Committee of Guangzhou General Hospital of Guangzhou Military Region and Zhongshan Hospital, Sun Yat-sen University. Written informed consent was obtained from all patients. Aortic specimens were collected from six type A TAD patients undergoing aortic replacement in the intimal tear position at the hospital in 2015. Normal thoracic aortas (NA) were obtained from six age- and gender-matched organ donors without aortic diseases. The specimens were immediately sliced into small pieces, frozen in liquid nitrogen, and preserved at -80 °C until further usage. For the circRNA microarray analysis, three TAD samples and three NA were randomly selected. The other six samples (three TAD tissues + three NA tissues) were prepared for qRT-PCR and Western blot analyses. The clinical characteristics of 12 patients were summarized in Supplementary Table 1.

### Total RNA isolation and quality control

Total tissue RNA was extracted from human TAD and NA tissues using TRIzol reagent (Invitrogen, NY, USA), according to the manufacturer’s instructions. The quality and concentration of RNA samples (Supplementary Table 2) were determined using the NanoDrop ND-1000 (Thermo Fisher Scientific, Wilmington, DE, USA). The genomic DNA (gDNA) contamination was excluded and the integrity of the total RNA were achieved by electrophoresis on a denaturing agarose gel (Supplementary Figure 1). The samples were preserved at -80 °C for validation experiments.

### RNA labeling and hybridization

Sample labeling and array hybridization were performed according to the manufacturer’s protocol (Arraystar Inc.). Briefly, total RNA was digested with RNase R (Epicentre, Inc.) to remove linear RNAs and enrich the circular RNAs. These circular RNAs were amplified and transcribed into fluorescent cRNA utilizing a random priming method (Arraystar Super RNA Labeling Kit; Arraystar). The labeled cRNAs (Supplementary Table 3) were hybridized onto the Arraystar Human circRNA Array V2.0 (8×15 K), with a total of 13,617 circRNA probes on the microarray. After washing the slides, the arrays were scanned using an Agilent G2505C Scanner. Agilent Feature Extraction software (version 11.0.1.1, USA) was used to analyze the acquired array images.

### circRNA microarray analysis

Agilent Feature Extraction software (version 11.0.1.1, USA) was used to analyze the acquired array images. Quantile normalization and subsequent data processing were performed using the R software (version 2.15). Low intensity filtering was performed after quantile normalization of the raw data and circRNAs that were found in at least three of the six samples and were flagged in “P” or “M” (“All Targets Value”) for further analysis. the log2-ratio was used for Quantile normalization. The differentially expressed circRNAs were selected according to the fold-change cut-off (fold change≥1.5) and P-value £0.05 or by the Volcano plot filtering. Hierarchical clustering was performed to distinguish the circRNA expression pattern among the samples. ‘Red’ indicates high relative expression, and ‘green’ indicates low relative expression.

### Quantitative real-time PCR (qRT-PCR)

Total RNA was isolated from TAD and NA tissues (three samples from each group, respectively) using TRIzol. For circRNA qRT-PCR analysis, out of 8 circRNAs, top 5 targeted miRNAs associated with TAD were selected for further investigation. These differential expressions were confirmed using qRT-PCR (in triplicate). Divergent primers (instead of commonly used convergent primers) were designed and optimized for the eight circRNAs. The sequence of circRNA results was acquired from the database “circBase” (http://circbase.mdc-berlin.de). For miRNA and mRNA analysis, total RNA was reverse transcribed using random primers. The relative expression of circRNAs and miRNA was determined using the 2^-ΔΔCt^ method by normalizing the expression against that of the housekeeping gene U6, whereas the mRNA expression levels were reported relative to that of GAPDH. Primers were synthesized by Saicheng Biotech (Guangzhou, China) and the sequences listed in Supplementary Table 4.

### Gene ontology (GO) and pathway analysis

We conducted Gene Ontology (GO) analysis (http://www.geneontology.org) to construct an annotation of genes and gene products in a wide variety of organisms. The gene functions encompassed physiological processes, molecular functions, and cellular components. The log10 (P-value) denoted the enrichment score representing the significance of GO term enrichment among differentially expressed genes. The KEGG pathway analysis determined the involvement of target genes in different physiological processes. Also, the log10 (P-value) represented the significant enrichment score of the pathway correlations.

### Identification of the circRNA-miRNA connectivity

The circRNA-miRNA connectivity was predicted with Arraystar’s home-made miRNA target prediction software based on TargetScan & miRanda. In order to establish circRNA-miRNA network, the Arraystar software was used to search MREs on the 262 differentially expressed circRNAs, which were selected the top 5 putative target miRNAs according to seed match sequences. Next , the defined group of miRNAs, which were previously described as differentially expressed in TAD [10, 11, 36-39], were used for a MRE sequence analysis. The map of circRNA-miRNA interaction network was illustrated by Cytoscape3.01.

### Prediction for circRNA-miRNA-mRNA network

Hsa_circ_101238 was used as a bait to enrich the circRNA-miRNA-mRNA network according to the miRNA target prediction GCBI (https://www.gcbi.com.cn/gclib/html/index) based on TargetScan coupled with miRanda. Cytoscape was employed to build a circRNA-miRNA-mRNA network of hsa_circ_101238.

### Luciferase reporter assay

Cells were seeded in 96-well plates at a density of 5×10^3^ cells per well 24 h before transfection. The cells were transfected with wild-type or mutated reporter vectors, miRNA mimics, miRNA inhibitor, and negative control. Lysates were harvested 24 h after transfection. Renilla luciferase activities were consecutively measured according to the dual-luciferase assay manual (Promega).

### Western blot

Protein was extracted using lysis buffer and the concentration determined by a bicinchoninic acid (BCA) protein assay kit. The extract was resolved on a 12% SDS-PAGE and transferred to PVDF membranes. After blocking for 2 h, the membranes were incubated with primary antibody against MMP9 (1:500, Santa Cruz Biotechnology; Santa Cruz, CA, USA) at 4 °C overnight, followed by appropriate HRP conjugated secondary antibody at room temperature for 2 h. The immunoreactive bands were visualized by ECL and normalized to β-actin used as an internal control.

### Statistical analysis

An unpaired Student’s t-test estimated the fold-changes that were filtered to describe the differentially expressed circRNAs in TAD. A robust detection was evaluated with the following definition for statistical significance: a probe was selected as differentially expressed with a P≤0.05 and a fold-change ≥1.5; a probe was selected as differentially repressed with a P≤0.05 and a fold change ≥-1.5. The false discovery rate(FDR) was calculated to correct the P-value on microarray analysis. All other statistical data were analyzed by GraphPad Prism 5.0 (GraphPad Software, La Jolla, CA, USA). The significance of qRT-PCR and Western blot between the TAD and the NA tissue groups was assessed by Student’s t-test and *P* < 0.05 was considered statistically significant.

## SUPPLEMENTARY MATERIALS FIGURES AND TABLES









## Abbreviations

TAD: thoracic aortic dissection
NA: normal donors without aortic diseases
ncRNAs: non-coding RNAs
miRNAs: microRNAs
MRE: miRNA response element
circRNAs: circular RNAs
ceRNA: competing endogenous RNAs
qRT-PCR: Quantitative real-time PCR
FLNA: filamin A
MMP9: matrix metalloproteinase 9
ECM: extracellular matrix
SMCs: smooth muscle cells
