# Supplementary material for: Circular RNA expression profile and potential function of hsa_circRNA_101238 in human thoracic aortic dissection
Source: Oncotarget. 2017 Jul 5;8(47):81825–37. doi: 10.18632/oncotarget.18998 (PMC5669851; doi:10.18632/oncotarget.18998)
Supplement: Supplementary file 1 [file oncotarget-08-81825-s001.pdf]

Circular RNA expression profile and potential function of hsa_ circRNA_101238 in human thoracic aortic dissection

Supplementary Material

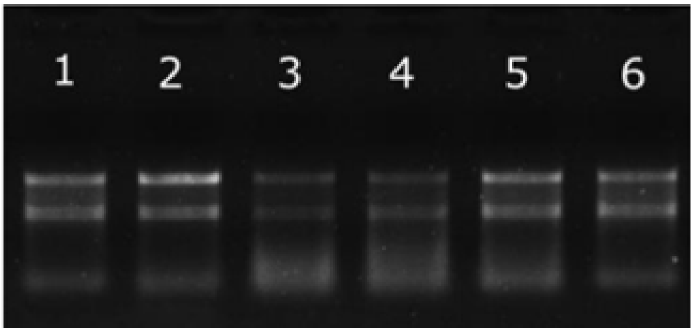

Supplementray Figure 1: RNA Integrity and gDNA contamination test by denaturing agarose gel electrophoresis
Lane 1: Total RNA of sample TAD1
Lane 2: Total RNA of sample TAD2
Lane 3: Total RNA of sample TAD3
Lane 4: Total RNA of sample NA1
Lane 5: Total RNA of sample NA2
Lane 6: Total RNA of sample NA3
The 28S and 18S ribosomal RNA bands should be distinct, intense bands. The intensity of the upper band should be 2-fold to that of the lower band. Small, diffused bands representing low molecular weight RNAs (tRNA and 5S ribosomal RNA) may be present. A diffuse smear of between 18S and 28S ribosomal bands, potentially comprised of mRNA and other heterogeneous RNA species. The DNA contamination in the RNA preparation as evident as a high molecular weight smear or band migrating above the 28S ribosomal RNA band. The degradation of the RNA will be reflected by the smears of the ribosomal RNA bands.

For Supplementray Tables 1,2,3,4,7 see in Supplementray Files
For Supplementray Tables 5,6 see in Excel Files Supplementray Files
