# Supplementary material for: Circular RNA expression profile and potential function of hsa_circRNA_101238 in human thoracic aortic dissection
Source: Oncotarget. 2017 Jul 5;8(47):81825–37. doi: 10.18632/oncotarget.18998 (PMC5669851; doi:10.18632/oncotarget.18998)
Supplement: Supplementary file 2 [file oncotarget-08-81825-s002.docx]

**Supplementary**

**Supplementary Table 1:** Clinical characteristics of TAD and NA groups

| TAD^a^ (n=3) NA^a^ (n=3) TAD^b^ (n=3) NA^b^ (n=3) |
| --- |
| Age (years) 49.3±5.6^*^ 41.6±3.1 49.0±4.3^*^ 42.3±4.1  Males/females 3/0 3/0 3/0 3/0  Hypertension 3 0 3 0  Atherosclerosis 0 0 0 0 |

TAD, thoracic aortic dissection; NA, normal donors without aortic diseases; ^*^P>0.05.
^a^Sample groups for circRNA microarray analysis.
^b^Sample groups for qRT-PCR and Western blot.

**Supplementary Table 2:** RNA quantification and quality assurance by NanoDrop ND-1000

| **Sample ID OD 260/280 OD 260/230 Conc Volume Quantity QC result**  **Ratio Ratio (ng/μL) (μL) (ng) Pass or Fail** |
| --- |
| TAD1 1.90 2.09 314.70 40 12588.0 Pass  TAD2 1.89 2.24 324.31 40 12972.4 Pass  TAD3 1.87 1.96 331.21 20 6624.2 Pass  NA1 1.89 2.17 323.45 40 12938.0 Pass  NA2 1.89 2.30 330.48 60 19828.8 Pass  NA3 1.93 1.89 326.56 60 19593.6 Pass |

*For spectrophotometer, the OD. A260 /A280 ratio should be close to 2.0 for pure RNA (ratios between 1.8 and 2.1 are acceptable). The OD. A260/A230 ratio should be more than 1.8.

**Supplementary Table 3: Labeling Efficiency**

| **Sample ID Dye Dye cRNA concentration Specific activity* Volume Total amount** **name pmol/μL (μg/μL) (pmol dye/μg cRNA) (μL) (μg)** |
| --- |
| TAD1 Cy3 16.39 0.70 23.33 10 7.03  TAD2 Cy3 16.28 0.72 22.56 10 7.21  TAD3 Cy3 16.77 0.71 23.57 10 7.11  NA1 Cy3 16.81 0.71 23.68 10 7.09  NA2 Cy3 16.66 0.74 22.55 10 7.38  NA3 Cy3 16.61 0.74 22.38 10 7.42 |

*For two colors, if the yield is <825 ng and the specific activity is <8.0 pmol Cy3 or Cy5/μg cRNA do not proceed to the hybridization step. Repeat cRNA preparation.

*For one color, if the yield is <1.65μg and the specific activity is <9.0 pmol Cy3 or Cy5/μg cRNA do not proceed to the hybridization step. Repeat cRNA preparation.

**Supplementary Table 4**: qRT-PCR primer sequences

| Primer name | Primer F (5'-3') | Primer R(5'-3') |
| --- | --- | --- |
| hsa_circRNA_101238 | CCAATAAAGCTGGTGCTAAAATAGGA | CTTGTTTAATGACTTTGGTGCCCT |
| hsa_circRNA_104634 | CCAGCAATGCAATCACCATAAAC | GATGGTGATGGAGATTTTGATGTG |
| hsa_circRNA_002271 | CAAACTGGAAAGCGTCTGTTTG | GCTCATTGGCCATTCAATAGG |
| hsa_circRNA_102771 | CGAACTAATTTCACCCCTTCTTCA | ACATCATCAATGCTGAGATGGAG |
| hsa_circRNA_104349 | CCTGCTACGAGATCTTGAATGC | ACCAAGCATAGGAGCCAAGG |
| hsa_circRNA_102683 | CAGGACAACGTGGAGAGAACTG | AGACTAACTGCAGATGGTTGCTGTAC |
| hsa_circRNA_005525 | GTCATTAGGCTGAGAATCCTCGTC | GTTGAACCAGAACAAAACCGAGTC |
| hsa_circRNA_103458 | CACTTGTCCCGCACATTCAC | GTGTGGGCACGAGGAGC |
| [hsa-miR-320a](file:///C:\Documents%20and%20Settings\Administrator\Application%20Data\Microsoft\李新忠%20H1512200%20AS-CR-005%20Human%20Circular%20RNA%20Microarray%20V2%2020160218-lyj\File%202.%20Data\Data%20Analysis%20Folder\circRNA_MREs\hsa-miR-320a_vs_hsa_circRNA_101238.pdf) | GTCGTATCCAGTGCAGGGTCCGAGGTATTCGCACTGGATACGACTCGCCC | CGGCAAAAGCTGGGTTGAGA |
| hsa-miR-138-5p  hsa-miR-593-5p | GGTGTCGTGGAGTCGGCAA  CACCAGCCAGGCATTGCTC | AACTTCACAACACCAGCTTA  CTCAACTGGTGTCGTGGA |
| U6 | CTCGCTTCGGCAGCACA | AACGCTTCACGAATTTGCGT |
| COL1A1 | CACCAATCACCTGCGGTACAGAA | CAGATCACGTCATCGCACAAC |
| COL6A3 | CCTAACCACATATGTTAGTGGAGGT | GAATGTCTCGCTTGCTCTCTG |
| FLNA | AGCCTCCACGAGACATCATC | CCAGTGTGTACTCCCCCTTG |
| GAPDH | GGGAAACTGTGGCGTGAT | GAGTGGGTGTCGCTGTTGA |

**Supplementary Table 5:** circRNAs expression profiling data.

**Supplementary Table 6:** Significant differentially expressed circRNAs (FC≥1.5 and P≤0.05) and the circRNA-microRNA interaction

**Supplementary Table 7:** **TAD-related target genes of the hsa_circRNA_101238**

| miRNA | common target symbol Target symbol | |
| --- | --- | --- |
| hsa-miR-320a  hsa-miR-138-5p  hsa-miR-593-5p | | MMP19 MMP9, ADAMTS5, COL11A2, TGFB2, TGFBR1, TGFBR2, TGFBR3,  COL1A1 SMAD4, SMAD6, MAPK14, IL10, TNF, HSP90AB1, HSPB3, BAK1,  SMAD2 CASP4, CASP7, CARD14  SMAD3 ADAMTS1, ADAMTS4, ADAMTS5, ADAMTS16, COL3A1, COL11A2,  SOD2 TGFB1, TGFBR3, SMAD4, HSP90AA1, HSPB7, BCL2, CASP7, CARD9,  CASP3 CARD11  FOSL2 MMP2, MMP11, MMP14, ADAMTS1, ADAMTS4, ADAMTS16, COL1A2, COL3A1, COL11A1, TGFB1, MAPK14, BCL2, BCL2L1, CARD16 |
